# Investigation of Oligoclonal IgG Bands in Tear Fluid of Multiple Sclerosis Patients

**DOI:** 10.3389/fimmu.2019.01110

**Published:** 2019-05-17

**Authors:** Martin W. Hümmert, Ulrich Wurster, Lena Bönig, Philipp Schwenkenbecher, Kurt-Wolfram Sühs, Sascha Alvermann, Stefan Gingele, Thomas Skripuletz, Martin Stangel

**Affiliations:** Departement of Neurology, Clinical Neuroimmunology and Neurochemistry, Hannover Medical School, Hannover, Germany

**Keywords:** multiple sclerois, oligoclonal band (OCB), tears, tear fluid, cerebrospinal fluid, CSF, MS

## Abstract

**Background:** Oligoclonal IgG bands (OCB) in the cerebrospinal fluid (CSF) represent a typical marker for inflammation in multiple sclerosis (MS) patients and have a predictive and diagnostic value in patients with a first suspected demyelinating event. The detection in tears remains controversial but some reports suggested a replacement of CSF analysis by OCB detection in tears. We aimed to investigate the value of OCB detection in tears systematically in patients with MS.

**Methods:** Tears of 59 patients with suspected or diagnosed MS were collected with Schirmer filter paper strips. Tear IgG was purified by affinity chromatography with protein G. After isoelectric focusing in polyacrylamide gels OCB detection was performed with direct silver staining. Paired triplets of CSF, serum, and tears were analyzed. For comparison purposes we additionally used other tear collection methods (flush procedure and plastic capillary tubes) or detection techniques (Immunoblotting). Clinical and paraclinical parameters are provided.

**Results:** IgG collection in tears was most reliable by using Schirmer strips. Thirteen patients had to be excluded due to insufficient sample material. Tear specific proteins that interfered with OCB detection were successfully eliminated by IgG purification. The concordance of OCB in tears and CSF of all investigated MS patients was 39% with a high rate of only marginal pattern in tears. Five patients demonstrated restricted bands in tears, neither detectable in CSF nor serum. Occurrence of OCB in tears was significantly associated with pathological visual evoked potentials (*P* = 0.0094) and a history of optic neuritis (*P* = 0.0258).

**Conclusion:** Due to the limited concordance, high rate of samples with insufficient material, and the unknown origin of tear IgG we cannot recommend that tear OCB detection may replace CSF OCB detection in MS patients. The detection of unique OCB in tears might offer new insights in ophthalmological diseases.

## Introduction

Oligoclonal IgG bands (OCB) detected in and restricted to the cerebrospinal fluid (CSF) are an immunological hallmark found in almost all patients diagnosed with multiple sclerosis (MS) (1, 2). The discovery of OCB dates back to the year 1959/1960, in which Lowenthal and his colleagues were able to detect a subdivided gamma globulin fraction in the CSF of various neuroinflammatory diseases for the first time (3, 4). The presence of OCB indicates a local B-cell response in the context of an inflammatory disease of the central nervous system (CNS). In MS patients, the detection of OCB is often the only way to identify intrathecal IgG synthesis. Its determination in patients with a first episode of neurological symptoms suggestive for a demyelinating disease gained in importance as a result of the 2017 revisions of the McDonald criteria (5). In addition, OCB in CSF are an important parameter for the risk assessment of the development of clinically definite multiple sclerosis when MS-typical symptoms occurred for the first time without fulfilling all diagnostic criteria for MS (6–9).

OCB detection in tears of MS patients was first demonstrated by Coyle and Sibony (10, 11). In their first study they found OCB in tears in 11 of 12 MS patients mostly with optic neuritis which were absent in normal controls (10). Possibly due to the poor resolution achieved by electrophoretic separation of proteins they were not able to distinguish the bands between tears and serum. Moreover, this study lacked a comparison with CSF. In their second study performed with isoelectric focusing and silver staining, 14 of 21 MS patients showed OCB in tear fluid (11). Clear distinction to serum was made in 18 MS patients, with two-thirds showing isolated OCB in tears. Only six tear samples were compared with CSF demonstrating dissimilar band patterns.

Three independent following studies with a total of 187 patients (84 MS patients, 65 other diseases, 23 healthy controls) using immunoperoxidase staining were not able to reproduce these results (12–14). First, Mavra et al. demonstrated one patient with neurosarcoidosis with OCB restricted to tears and CSF. All others, especially 28 patients with MS and 4 with optic neuritis, showed no OCB in tears (12). Second, Liedtke et al. were able to detect OCB in tears in 3 of 38 MS patients (13). Precise data if these samples had matched bands in CSF or serum are missing. Third, Martino et al. revealed one MS patient with unique OCB in tears with no correspondence in the paired CSF and serum (14). All other 17 patients with MS and 17 other neurological patients displayed no OCB in tears.

The most recent studies, all published by the same group, revealed a concordance rate of 73% (27 of 37 patients) and 80% (48 of 60 patients), respectively, for the presence or absence of OCB in tears and CSF in MS patients (15, 16). Interestingly, surprisingly low 81% (30 patients) or 75% (45 patients), respectively, of all MS patients had positive OCB in CSF. The concordance rate between CSF and tears in patients with positive OCB in CSF (CSF^OCB+^) was 22 of 30 patients (73%) and 43 of 45 patients (96%), respectively. The same group analyzed tears of 69 patients with clinically isolated syndrome (CIS) with a concordance of 78% (54 of 69 patients) for OCB status in tears and CSF OCB. The concordance rate between CSF and tears in CSF^OCB+^ patients was 29 of 44 patients (66%) (17). This study was criticized because of methodological shortcomings (18). Another investigation from this group in 42 patients with radiologically isolated syndrome (RIS) showed—similar to their second MS study—in all CSF^OCB+^ patients a concordance rate of OCB between tears and CSF of 96% (21 of 22 patients) (19). All studies investigating OCB in tears, their methodology and results are summarized in Table 1.

**Table 1:** Studies investigating OCB in tears.

**Study**	**Country**	**Study population**	**Methodology (collection technique, gel type, separation technique, IgG visualization, stimulation of tearing)**	**Results**
Coyle and Sibony (10)	USA	12 MS 20 controls	Glass capillary tubes, SDS-polyacrylamide gel, electrophoresis, silver staining, stimulation by onions/aromatic ammonia	OCB in tears from the involved eye in 4 patients with acute optic neuritis faint OCB in tears in 7 patients, 5 of them with history of optic neuritis no OCB in tears of 13 controls, no CSF-matching done
Coyle et al. (11)	USA	24 MS 20 OD 15 controls	Glass capillary tubes, agarose gel, IEF, silver staining, stimulation by onions/aromatic ammonia	OCB in tears in 14 of 21 MS patients (“most not present in serum”) OCB in tears in 1 of 15 not neurological patients also present in serum (type 4) no OCB in tears of 11 controls, CSF OCB data for 6 MS patients
Mavra et al. (12)	UK	28 MS 4 ON 30 OD	Glass capillary tubes, agarose gel, IEF, immunoperoxidase staining, stimulation by onions	no OCB in tears of any MS/ON patient OCB in tears in 1 of 30 other patients (type 2; neurosarcoidosis) CSF data for all but 8 patients
Liedtke et al. (13)	Germany	38 MS 14 OD 23 controls	^*^Schirmer strip or capillary tubes, polyacrylamide gel, IEF, immunoperoxidase staining, stimulation by ammonia vapor in case of capillary tubes	no OCB in tears in 35 of 38 MS patients no OCB in tears in 0 of 13 other patients no OCB in tears in 19 of 21 controls only 17 cases with paired CSF and serum samples, not clearly assigned
Martino et al. (14)	Italy	18 MS 17 OD	Glass capillary tubes, agarose gel, IEF, immunoperoxidase staining, stimulation by warm air flow	no OCB in tears in 16 of 18 MS patients (94% CSF^OCB+^), 1 MS patient with unique OCB in tears, 1 MS patient with OCB in tears also present in serum (type 4) OCB in tears in 3 of 17 other patients also present in serum (type 4)
Forzy et al. (15)	France	66 MS 55 OD	Schirmer strip, agarose gel, IEF, silver staining, no stimulation	27 of 37 MS patients with same result for OCB in tears and CSF (81 % CSF^OCB+^), 29 MS patients without CSF-matching
Devos et al. (16)	France	63 MS 52 OD 13 OIND	Schirmer strip, agarose gel, IEF, silver staining, no stimulation	48 of 60 MS patients with same result for OCB in tears and CSF (75% CSF^OCB+^) 44 of 50 OD patients with same result for OCB in tears and CSF (8% CSF^OCB+^) 10 of 13 OIND patients with same result for OCB in tears and CSF (31% CSF^OCB+^) (exclusion of 5 patients because of positive OCB in serum)
Calais et al. (17)	France	82 CIS	Schirmer strip, agarose gel, IEF, immunoperoxidase staining, no stimulation	54 of 69 CIS patients with same result for OCB in tears and CSF (64% CSF^OCB+^) (exclusion of 13 patients because of sample dilution)
Lebrun et al. (19)	France	45 RIS	Schirmer strip, agarose gel, IEF, immunoperoxidase staining, no stimulation	41 of 42 RIS patients with same result for OCB in tears and CSF (52% CSF^OCB+^) (exclusion of 3 patients because of insufficient material)

*MS, multiple sclerosis; ON, optic neuritis; OD, other disease or condition; OIND, other inflammatory neurological disease; CIS, clinically isolated syndrome; RIS, radiologically isolated syndrome; IEF, isoelectric focusing; CSF^OCB+^, evidence of OCB in CSF without corresponding OCB in paired serum. In (10–13) only incomplete or missing data on paired triplets of tears, CSF and serum exists. In (10–15) clear data to patient dropout because of missing material is lacking*.

* *Liedtke et al. refer to another publication for collection technique and stimulation of tearing describing two different methods (20). Type 4 defines a negative OCB pattern (21)*.

In summary, two independent research groups showed a relevant proportion of isolated bands in paired tear/CSF samples, whereas three independent research groups were unable to confirm these results. The authors of most recent positive studies suggested to partially replace CSF OCB detection by tear OCB detection in MS, CIS, and RIS patients (16, 17, 19). Because of the invasive character of a lumbar puncture tear collection might be a promising non-invasive tool to detect OCB in patients who decline a lumbar puncture, undergo follow-up analysis, or have anatomical or medical reasons why lumbar puncture is not possible. The aim of this study was to prove the reliable detectability of OCB in tears of MS patients by isoelectric focusing, silver staining and tear IgG purification.

## Patients and Methods

### Patient Characteristics

Patients with suspected or diagnosed multiple sclerosis were recruited at the Department of Neurology at Hannover Medical School, Germany. The study was approved by the local ethics committee (No. 7218) and all patients gave written consent before enrollment. To minimize the risk of artificial results patients with infectious eye disease or treatment with tear-reducing drugs were excluded from the study. In total, 119 tear samples were collected from 59 different patients with suspected or diagnosed MS. Final diagnosis of MS/CIS was made in 28 patients (Table 2) according to the McDonald criteria (2017) (5).

**Table 2:** Patient characteristics and CSF OCB status.

**Group of diseases**		**Patients n**	**Age, years range (median)**	**Sex f:m (percentage)**	**Positive CSF OCB n (percentage)**
All		59	18–69 (37)	41: 18 (70: 30)	37 (63)
MS	RRMS SPMS	22 2	18–57 (35) 55; 61	14: 8 (64: 36) 2: 0 (100: 0)	21 (96) 2 (100)
CIS		4	20 – 55 (39)	3: 1 (75: 25)	3 (75)
OIND	Autoimmune (a) Infectious (i)	11 2	26–69 (38) 32; 52	9: 2 (82: 18) 1: 1 (50: 50)	10 (91) 1 (50)
OI		4	26–39 (37)	4: 1 (100: 0)	0 (0)
Control		14	19–68 (42)	8: 6 (57: 43)	0 (0)

*RRMS, relapsing-remitting multiple sclerosis; SPMS, secondary progressive multiple sclerosis; CIS, clinically isolated syndrome; OINDa/i, other inflammatory neurological disease [relapsing opticus neuritis, neuromyelitis optica, myelitis, MOG positive encephalomyelitis, (primary) angiitis of the central nervous system, papillitis, viral meningitis]; OI, (possible) subclinical or clinical ocular inflammation; Control group [spinal muscular atrophy, amyotrophic lateral sclerosis, myopathy, unspecific paresthesia / hypesthesia / vertigo / muscular cramps / (back) pain / gait abnormality]. Positive OCB are defined as pattern type 2/2a or type 3/3a*.

### Sample Collection and Preanalytical Preparation

CSF was obtained by lumbar puncture and immediately analyzed in the neurochemistry laboratory of the Department of Neurology as reported previously (22). Successful demonstration of OCB in tears from MS patients in the most recent reports relied on collection of tears with Schirmer filter paper strips (15–17, 19). This method was mainly used in our study. Tears were collected from the conjunctival sac of the lateral inferior eyelid until the Schirmer strip was completely wetted or for a maximum of 5 min (23). Time and wetting length were recorded. The unwet parts were cut off and the wet parts were instantly placed separately in small plastic vials on ice. Tears were separated from the paper strips by centrifugation (60 s, 12 100 g, temperature: 20°C). In order to increase tear extraction from the Schirmer strip, it was moistened with 50 μL Ringer's solution and centrifuged again under the same conditions after 1 min. The samples were stored at −80°C until further analysis. In addition to the sampling with Schirmer test we also used a “flush” procedure (24). Irrigation of the ocular surface with 50 μl 0.9% saline yields a higher volume and is more comfortable to the patient. Plastic capillary tubes were used for collection of tears in a few patients. No stimulation of tearing was provoked in order to avoid artificial changes of the tears. In some patients suffering from a sicca syndrome it was impossible to gather enough fluid for the investigation.

### Sample Measures

In all samples total protein content was determined by the Coomassie Blue method (25). Serum and CSF were adjusted to 20 mg/l IgG and placed side by side with an appropriately diluted tear sample of the same patient. Isoelectric focusing was performed on polyacrylamide gels (pH 6–10). Direct silver staining as well as immunoblotting were applied for the detection of OCB. Following the recommendations of the European consensus on CSF analysis in MS positive OCB are defined as pattern type 2 or type 3 (21). CSF with more than 1 but < 4 CSF restricted OCB is defined as type 2a or type 3a. Negative OCB are defined as pattern type 1 or type 4. The same nomenclature referring to serum was used for OCB in tears.

### Tear IgG Purification

In contrast to CSF, tears contain a high number of basic proteins (lactoferrin, lysozyme, cystatin C) in high concentrations (26). These proteins interfere with OCB detection both in direct silver staining and in immunoblotting by masking a considerable portion of the migration path. Removal of the alkaline tear proteins and isolation of pure IgG was achieved by prior affinity chromatography with protein G (Protein G Mag Sepharose®, GE Healthcare, UK).

### Statistics

Results were analyzed with GraphPad Prism 5.02 (GraphPad Software, USA). Fisher's exact test was used to measure the independence of two categorical variables. This test offers an exact test result even for small sample sizes. *P* < 0.05 were considered as statistically significant.

## Results

**Figure 1:**
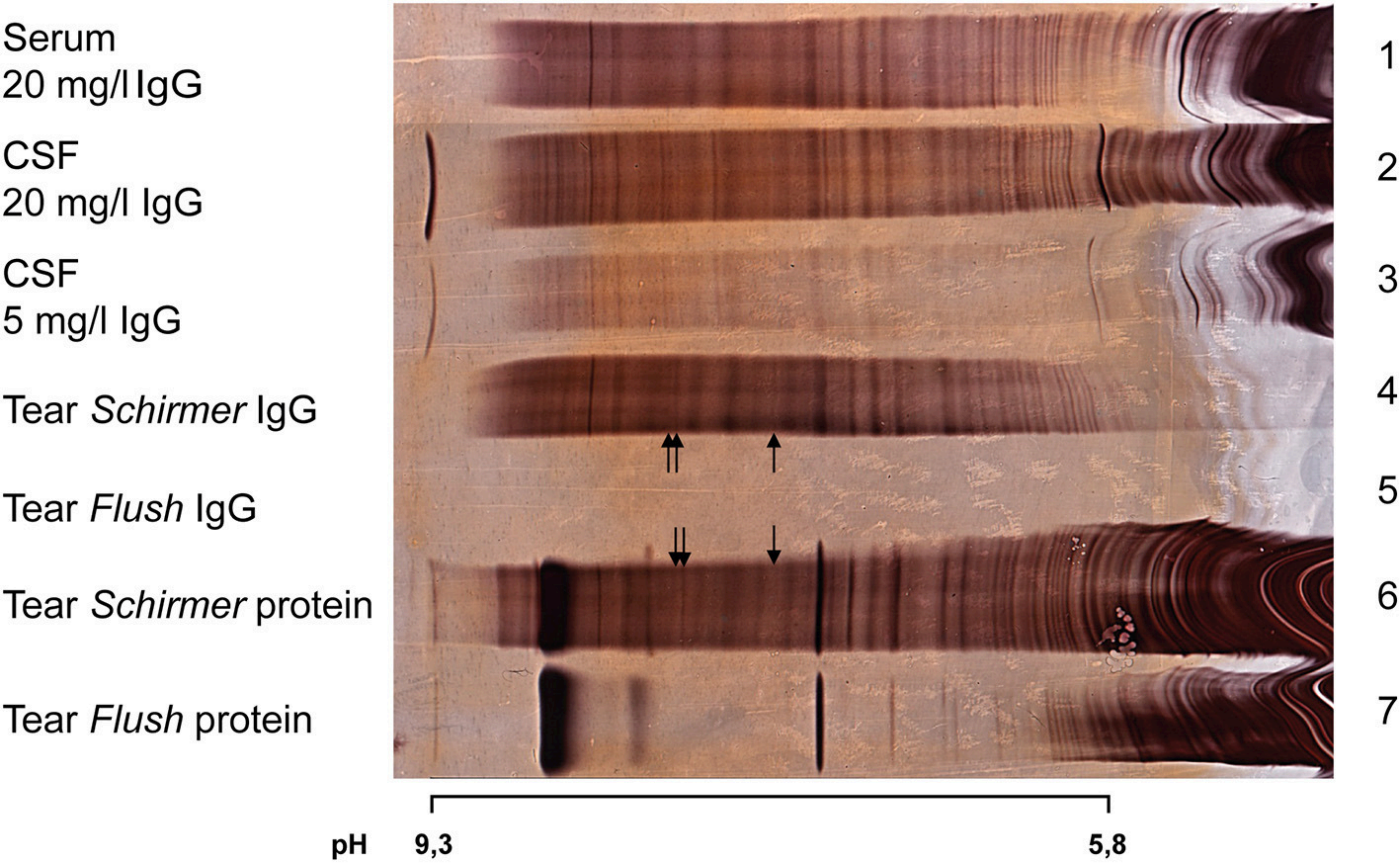
Silver staining after isoelectric focusing on polyacrylamide gel of serum, CSF, and tear fluid (Schirmer or Flush method) of a patient diagnosed with relapsing-remitting MS. Arrows indicate positive OCB in tears. In the lanes with “tear Schirmer/Flush IgG” the IgG was isolated by protein G affinity purification prior to electrophoresis, while in the lanes “tear Schirmer/Flush protein” the unpurified sample was used.

In most tears collected with either the flush or the capillary tube method the concentration of IgG was below the detection level (Figure 1, lane 5 and 7). Tear collection with the Schirmer filter paper strips yielded better results and thus the reported results are based on tear samples gained by Schirmer strips.

From the 59 recruited patients, 13 patients (22%) were excluded because of a lack of sufficient material. The tears of 3 patients were used for immunoblotting. Of the remaining 43 patients, 12 (28%) had OCB in CSF and tears, in 14 patients (32%) OCB were only detectable in CSF, and 12 patients (28%) showed OCB neither in CSF nor in tears (for details see Table 3A). Interestingly, in 5 patients (12%) we found positive OCB in tears without OCB detection in CSF. Of note 10 of 17 patients with positive OCB in tears showed only a marginal OCB pattern (type 2a or 3a). One of these patients diagnosed with relapsing-remitting MS with a characteristic positive OCB pattern in CSF (type 2) and few detectable OCB in tears (type 2a) is shown in Figure 1. Of the 3 samples stained by immunoblotting we were able to verify oligoclonal IgG in CSF but had inconclusive results in tears (Table 3A).

**Table 3A:** OCB results and clinical data of all recruited patients.

**Disease**	**Pat**.	**OCB**		**Remarks**	**Patient characterization (clinical data)**							
	**#**	**CSF**	**tears**		**Age**	**Sex**	**Symptoms at time of investigation**	**IM**	**ID**	**EDSS**	**Previous neurological medication**	**Current neurological medication**
**RRMS**	1	**2**	n. d.		22	f	Hypesthesia, paresis	01/2012	01/2012	3.0	GLAT, DMF, NAT	ALZ
	2	**2**	**2**		46	f	Paresis	11/2015	12/2015	3.5	DMF	DMF
	3	**2**	**2a**	External CSF	32	m	Hypesthesia, paresis	2009	2011	1.5	INF, DMF	ALZ
	4	**2**	1		49	m	Paresis	1999	1999	6.5	AZA, INF, GLAT, NAT, FTY, DAC	none
	5	**2**	**2a**		36	m	Optic neuritis (left)	08/2017	08/2017	1.5	none	none
	6	**2**	**2a**		27	m	Optic neuritis (left)	06/2017	08/2017	1.0	none	none
	7	**2**	1		26	f	Hypesthesia, paresis	11/2014	12/2014	3.0	DMF	ALZ
	8	**2**	n. d.	Not enough material	57	f	Optic neuritis (right)	1981	09/2017	3.0	none	none
	9	**2**	n. d.	Not enough material	47	f	Hypesthesia, paresthesia, paresis	2013	09/2015	1.0	none	DMF
	10	**2**	1		43	f	Trigeminal neuralgia	10/2017	11/2017	1.0	none	none
	11	**2**	1		36	f	Optic neuritis (links)	09/2015	07/2016	1.0	INF	DMF
	12	**2**	**2**		44	m	Facial palsy	1998	2014	2.5	DMF, INF, FTY	ALZ
	13	**2**	**2**	CFS during natalizumab	20	f	Hypesthesia	12/2012	01/2013	3.0	GLAT, FTY, NAT	ALZ
	14	**2**	1		33	f	Paresis	2010	2010	4.0	INF, GLAT, NAT, DAC	none
	15	**2**	**2a**		37	m	Optic neuritis (right)	04/2018	04/2018	2.0	none	none
	16	**2**	1		33	f	Optic neuritis (left)	04/2018	04/2018	2.0	none	none
	17	1	**2a**	External CSF, discrepant	29	f	Optic neuritis (left)	06/2015	02/2019	1	none	none
	18	**2**	1		30	m	Paresthesia	12/2016	07/2017	1.0	none	none
	19	**2**	1		18	f	Paresthesia	12/2016	07/2017	1.0	none	none
	20	**2**	inc.	Blot	28	m	Paresthesia	08/2017	09/2017	1.0	none	none
	21	**2**	1	Tears pooled from 2 days	50	f	Diplopia	09/2017	09/2017	1.0	none	none
	22	**2**	1		45	f	Optic neuritis (right)	09/2017	09/2017	1.0	none	none
**SPMS**	1	**2**	n. d.	Not enough material	61	f	Paresis, bladder dysfunction	1983	1983	7.5	Mitoxantrone	IVMP
	2	**2**	n. d.	Not enough material	55	f	Paresis, bladder dysfunction	03/2000	03/2000	6.0	GLAT, NAT	Mitoxantrone
**CIS**	1	4	4		53	f	Optic neuritis (left)	07/2017	07/2017	3.0	none	none
	2	**2**	n. d.	Not enough material	24	f	Optic neuritis (right)	08/2017	08/2017	3.0	none	none
	3	**2**	n. d.	Not enough material	55	f	Optic neuritis (right)	08/2017	08/2017	3.0	none	none
	4	**2**	inc.	Blot	20	m	Optic neuritis (right)	08/2017	08/2017	1.0	none	none
**OINDa**	1	**3**	**3a**		51	f	Vertigo, paresthesia	06/2017	07/2017	2.0	none	none
	2	**3**	4		69	f	Hypesthesia, paresis, bladder dysf.	07/2017	07/2017	5.0	none	none
	3	**2**	1		26	m	Hypesthesia, paresis	06/2017	08/2017	n. a.	none	none
	4	**2a**	1		26	f	Hypesthesia	08/2017	n. a.	n. a.	none	none
	5	1	inc.	Blot	45	f	Hypesthesia, paresthesia	05/2017	08/2017	1.5	none	none
	6	**2**	n. d.	Not enough material	29	f	Optic neuritis (right)	11/2017	11/2017	1.0	none	none
	7	**2**	**2**	Replapsing ON	31	f	Optic neuritis (right)	05/2017	06/2017	1.0	none	Rituximab
	8	**2**	**2a**		49	f	Paresthesia	02/2018	n. a.	n. a.	none	none
	9	**3**	n. d.	Not enough material	42	f	Headache, hypesthesia	2016	n. a.	n. a.	none	none
	10	**3**	**3**		38	m	Unspecific	03/2018	n. a.	n. a.	none	none
	11	**2**	**2**		27	f	Seizure	03/2019	n. a.	n. a.	none	none
**OINDi**	1	4	1		52	f	Loss of vision	09/2017	n. a.	n. a.	none	none
	2	**2**	1		32	m	Paresthesia	03/2018	n. a.	n.a	none	none
**OI**	1	1	**2a**	Discrepant	36	f	Optic neuritis (right)	05/2014	05/2014	3.0	none	none
	2	1	**2**	Discrepant, relapsing Zoster	37	f	Paresthesia	2010	n. a.	n. a.	none	none
	3	4	**3a**	Discrepant	39	f	Hypesthesia	12/2017	n. a.	n. a.	none	none
	4	4	**3a**	Discrepant, Zoster V1	26	f	Painful skin changes	03/2019	n. a.	n. a.	none	none
**Control**	1	1	n. d.	Not enough material	46	f	Aura	08/2017	n. a.	n. a.	none	none
	2	4	n. d.	Not enough material	47	m	Unspecific gait abnormality	2015	n. a.	n. a.	none	none
	3	1	1		48	f	Paresis	2015	n. a.	n. a.	none	none
	4	1	1		27	f	Unspecific vertigo	03/2019	n. a.	n. a.	none	none
	5	1	1		50	f	Muscular cramps	2017	n. a.	n. a.	none	none
	6	1	1		34	m	Pain	02/2019	03/2019	n. a.	none	none
	7	4	n. d.	Not enough material	49	m	Back pain	2012	n. a.	n. a.	none	none
	8	1	1		37	f	Hypesthesia	03/2019	n. a.	n. a.	none	none
	9	1	1		55	f	Paresthesia	03/2019	n. a.	n. a.	none	none
	10	1	1		36	f	Paresis	1992	1992	n. a.	none	Nusinersen
	11	1	1		68	m	Paresis	2017	03/2019	n. a.	none	none
	12	1	n. d.	Not enough material	19	m	Paresis	2016	2016	n. a.	none	Nusinersen
	13	1	1		22	m	Paresis	1998	1998	n. a.	none	Nusinersen
	14	1	1		23	f	Hypesthesia	03/2019	n. a.	n. a.	none	none

*EDSS, expanded disability status scale; f, female; ID, initial diagnosis; IM, initial clinical manifestation; m, male; n. a., not applicable; ON, optic neuritis; MS medication: Alemtuzumab (ALZ), Azathioprine (AZA), Daclizumab (DAC), Dimethyl Fumarate (DMF), Fingolimod (FTY), Glatiramer Acetate (GLAT), Interferon-Beta (INF), intravenous methylprednisolone (IVMP), Natalizumab (NAT). Positive OCB are defined as pattern type 2/2a or type 3/3a. Negative OCB are defined as pattern type 1 or type 4. Bold entries indicate a pathology*.

**Figure 2:**
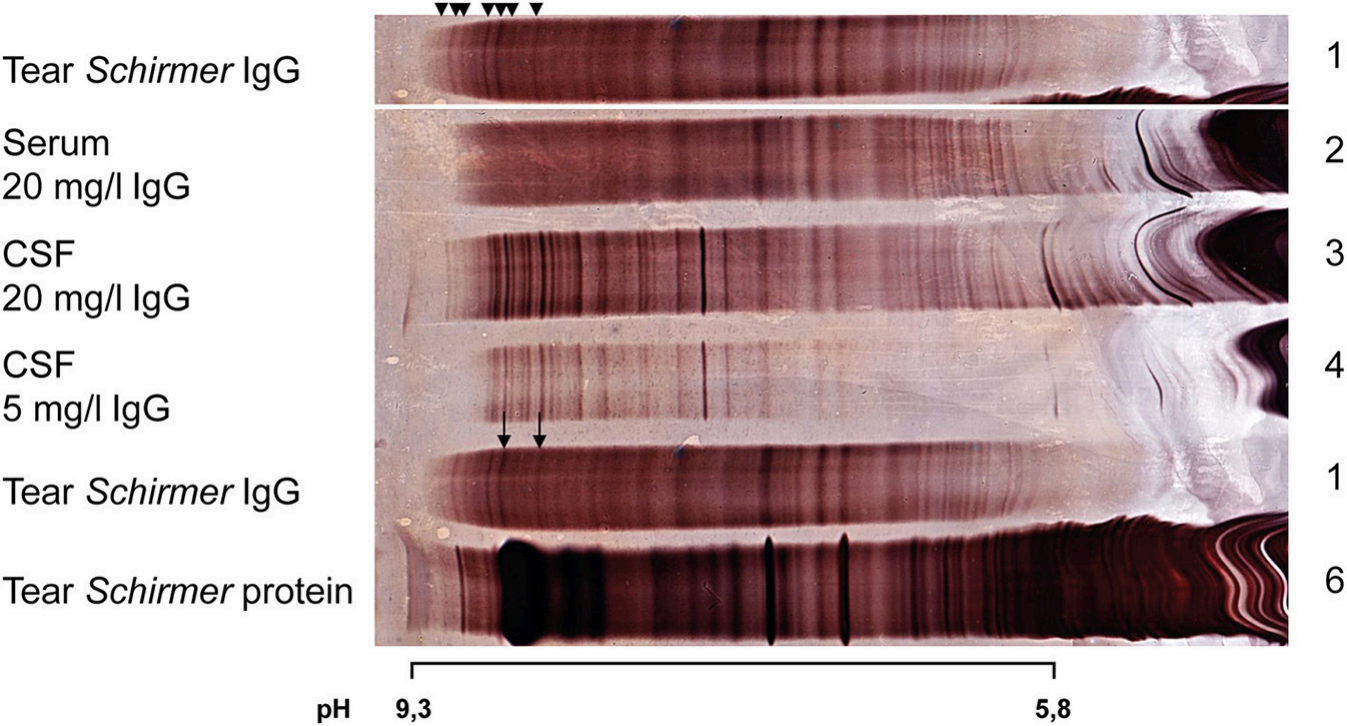
Silver staining after isoelectric focusing on polyacrylamide gel of serum, CSF, and tear fluid (Schirmer method) of a patient diagnosed with MOG-IgG positive encephalomyelitis. For a better overview lane 1 (Tear *Schirmer* IgG) is duplicated. Arrowheads indicate bands in tear fluid, at least 3 of which were unique bands in tears. Arrows indicate 2 positive OCB in tears comigrating with CSF OCB. The comparison between lane 1 (sample with purified IgG) and lane 6 (unpurified sample) illustrates the interference of tear specific protein with OCB in the cathodic section of the gel.

Figure 2 demonstrates the characteristic problem of OCB detection in unprepared tears: A relevant cathodic part of the gel is covered by tear specific proteins (see Figure 2, lane 6). IgG purification by affinity chromatography with protein G eliminates these alkaline proteins (see Figure 2, lane 1). The great advantage of this approach is that the silver stain could be used directly for the demonstration of IgG without interference from other proteins.

**Figure 3:**
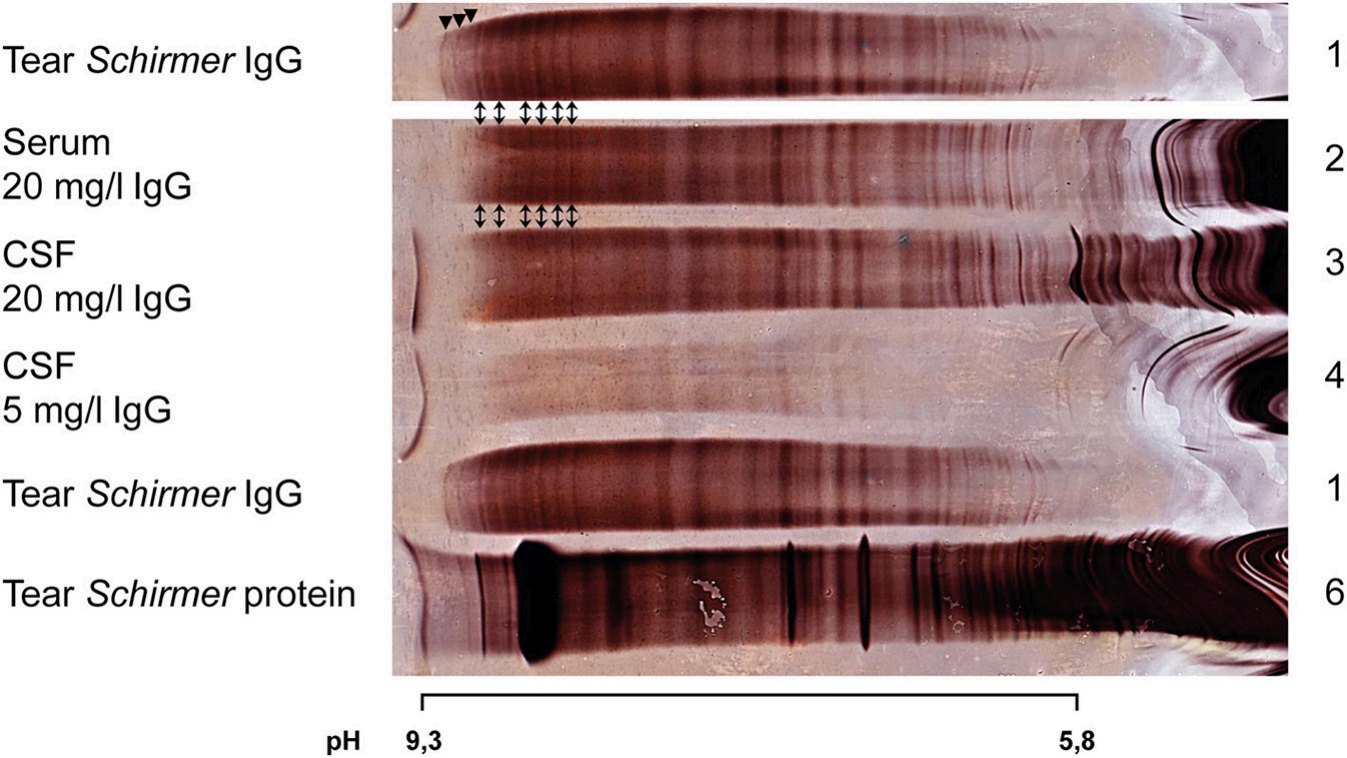
Silver staining after isoelectric focusing on polyacrylamide gel of serum, CSF, and tear fluid (Schirmer method) of a patient suffering from unspecific paresthesia. For a better overview lane 1 (Tear *Schirmer* IgG) is duplicated. Arrowheads indicate unique bands in tears. Double arrows indicate 6 identic OCB in paired tears, serum, and CSF.

Figure 3 illustrates a patient with unique OCB in tears suffering from unspecific complaints.

**Figure 4:**
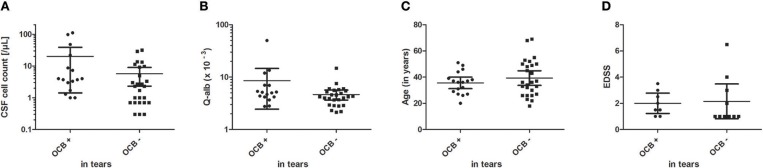
Influence of CSF cell count **(A)**, Q-alb **(B)** and age **(C)** of all patients and EDSS **(D)** of all MS patients on the occurrence of OCB in tear fluid. For better visualization, a logarithmic illustration was used in **(A)** and **(B)**. The mean with 95% confidence interval is shown.

Pathological visual evoked potentials showed a highly significant association to the occurrence of OCB in tears (*P* = 0.0094). In addition, a history of optic neuritis was significantly associated with OCB in tears (*P* = 0.0258). Other clinical or paraclinical factors had no influence on the occurrence of OCB in tears (CSF cell count: *P* = 1.0; blood-brain barrier dysfunction: *P* = 0.0608; cell profile: *P* = 1.0; MRZ reaction: *P* = 1.0; magnetic resonance imaging of the brain: *P* = 1.0; magnetic resonance imaging of the spinal cord: *P* = 0.6699; sex: *P* = 0.7357; for details see Tables 3A,B and Figures 4A,B). Neither the age of all patients nor the EDSS of MS patients influenced the occurrence of OCB in respective tears (Figures 4C,D). Contradicting, one patient suffering from optic neuritis without visual evoked potential alterations but matching magnetic resonance imaging and very recently diagnosed with MS showed OCB in tears but not in CSF (for details see Table 3B, Pat. # 17, RRMS disease group). However, since the OCB investigation was carried out in an external laboratory, it cannot be excluded that the lack of OCB in CSF is due to insufficient sensitivity of OCB detection.

**Table 3B:** OCB results and paraclinical data of all recruited patients.

**Disease**	**Pat**.	**OCB**		**Patient characterization (paraclinical data)**							
	**#**	**CSF**	**tears**	**CC**	**Q-alb**	**CSF cell profile**	**MRZ**	**cMRI**	**sMRI**	**VEP**	**ever ON**
**RRMS**	1	**2**	n. d.	**20**	-	Normal	**pos**.	**Multiple T2-h. lesions**	**Multiple T2-h. lesions**	n. d.	**yes**
	2	**2**	**2**	3.7	5.0	Normal	neg.	**Multiple T2-h. lesions including the left optic nerve**	normal	**Prolonged latencies left**	**yes**
	3	**2**	**2a**	n. d.	n. d.	n. d.	n. d.	**Multiple T2-h. lesions**	**Multiple T2-h. lesions**	**Prolonged latencies right**	**yes**
	4	**2**	1	0.3	5.79	Normal	n. d.	**Multiple T2-h. lesions**	**Multiple T2-h. lesions**	Normal	no
	5	**2**	**2a**	**7.3**	4.35	**Plasma cells**	neg.	**Multiple T2-h. lesions**	n. d.	**Decrease in left amplitude**	**yes**
	6	**2**	**2a**	**8.7**	5.0	**Plasma cells**	neg.	**T2-h. lesion with CE of the left optic nerve**	**Some T2-h. lesions**	**Prolonged latencies left**	**yes**
	7	**2**	1	0.3	4,9	Normal	neg.	**Multiple T2-h. lesions**	**Multiple T2-h. lesions**	Normal	**yes**
	8	**2**	n. d.	4.7	3.62	**act. monocytes**	**pos**.	**Multiple T2-h. lesions**	n. d.	**Decrease in left amplitude**	**yes**
	9	**2**	n. d.	**34**	4,18	**Plasma cells**	**pos**.	**Multiple T2-h. lesions**	n. d.	n. d.	no
	10	**2**	1	1.0	4.24	Normal	neg.	**Multiple T2-h. lesions, one with CE**		Normal	no
	11	**2**	1	2.3	2.20	**Plasma cells**	**pos**.	**Multiple T2-h. lesions including the left optic nerve**	n. d.	**Prolonged latencies left**	**yes**
	12	**2**	**2**	3.7	**11.97**	Normal	neg.	**Multiple T2-h. lesions, one with CE**	Normal	n. d.	no
	13	**2**	**2**	**98.0**	4.66	**act. lymphocytes**	**pos**.	**Multiple T2-h. lesions**	**Multiple T2-h. lesions**	**Prolonged latencies left**	**yes**
	14	**2**	1	1.0	6.01	Normal	neg.	**Multiple T2-h. lesions**	**LETM, CE**	**Prolonged latencies left**	**yes**
	15	**2**	**2a**	**22.0**	5.14	**Plasma cells**	**pos**.	**Multiple T2-h. lesions including right optic nerve**	Normal	**Prolonged latencies right**	**yes**
	16	**2**	1	**11.3**	4.62	**Plasma cells**	neg.	**Multiple T2-h. lesions including left optic nerve**	Normal	Normal	**yes**
	17	1	**2a**	4.0	5.20	normal	n. d.	**Some T2-h. lesions including left optic nerve**	**One T2-h. lesions**	Normal	yes
	18	**2**	1	3.0	3.93	Normal	**pos**.	**Multiple T2-h. lesions**	n. d.	Normal	no
	19	**2**	1	**29**	2.14	**Plasma cells**	neg.	**Some T2-h. lesions**	n. d.	Normal	no
	20	**2**	inc.	2.7	5.20	Normal	neg.	**One T2-h. lesion**	**One T2-h. lesions with CE**	n. d.	no
	21	**2**	1	2.7	5.26	**act. lymphocytes**	**pos**.	**Some T2-h. lesions**	n. d.	n. d.	no
	22	**2**	1	**5.0**	2.30	**Plasma cells**	neg.	**Multiple T2-h. lesions including both optic nerves**	n. d.	**Prolonged latencies right**	**yes**
**SPMS**	1	**2**	n. d.	**8.0**	n. d.	**Plasma cells**	neg.	n. d.	n. d.	n. d.	n. d.
	2	**2**	n. d.	3	n. d.	n. d.	n. d.	**Multiple T2-h. lesions**	n. d.	**Prolonged latencies right**	**yes**
**CIS**	1	4	4	0.3	3.69	Normal	neg.	Normal	Normal	**Prolonged latencies left**	**yes**
	2	**2**	n. d.	**20.3**	3.49	**Plasma cells**	**pos**.	**T2-h. lesion with CE of the right optic nerve**	n. d.	**Cortical signal loss right**	**yes**
	3	**2**	n. d.	**61.3**	4.89	**act. lymphocytes**	neg.	**T2-h. lesion of the right optic nerve**	n. d.	**Cortical signal loss right**	**yes**
	4	**2**	inc.	**10.3**	3.08	**Plasma cells**	**pos**.	**T2-h. lesions with CE of the right optic nerve**	n. d.	**Prolonged latencies right**	**yes**
**OINDa**	1	**3**	**3a**	**111**	**13.5**	**act. lymphocytes**	neg.	**Multiple T2-h. lesions**	**Multiple T2-h. lesions**	**Prolonged latencies right**	**yes**
	2	**3**	4	**9.3**	**14.90**	**Plasma cells**	n. d.	**Multiple T2-h. lesions**	**LETM, CE**	n. d.	no
	3	**2**	1	**9.7**	4.56	**Plasma cells**	neg.	**DWI hyperintensities**	n. d.	n. d.	no
	4	**2a**	1	3.3	3.72	**act. lymphocytes**	neg.	Normal	n. d.	Normal	no
	5	1	inc.	1.3	5,67	Normal	neg.	Unspecific	**One T2-h. lesions**	n. d.	no
	6	**2**	n. d.	**10.3**	4.49	**act. lymphocytes**	neg.	**Multiple T2-h. lesions**	**Multiple T2-h. lesions with CE**	n. d.	**yes**
	7	**2**	**2**	4.0	**7.0**	**Plasma cells**	neg.	**Multiple T2-h. lesions**	Normal	**Prolonged latencies right**	**yes**
	8	**2**	**2a**	1.3	3.67	Normal	n. d.	Normal	n. d.	n. d.	no
	9	**3**	n. d.	1.3	4.15	**Siderophages**	neg.	Unspecific	n. d.	n. d.	no
	10	**3**	**3**	**48.0**	**6.95**	**Plasma cells**	neg.	Unspecific	n. d.	n. d.	no
	11	**2**	**2**	1.7	2.81	Normal	neg.	Unspecific	n. d.	n. d.	no
**OINDi**	1	4	1	**13.3**	5.50	Normal	neg.	Unspecific	n. d.	Normal	no
	2	**2**	1	**13.7**	4.22	**Plasma cells**	n. d.	Unspecific	n. d.	n. d.	no
**OI**	1	1	**2a**	1.0	4.17	Normal	neg.	Normal	Normal	n. d.	**yes**
	2	1	**2**	1.0	2.76	Normal	neg.	Normal	n. d.	n. d.	no
	3	4	**3a**	3.3	5.35	Normal	neg.	Unspecific	n. d.	n. d.	no
	4	4	**3a**	3.0	4.19	Normal	n. d.	Normal	n. d.	n. d.	no
**Control**	1	1	n. d.	2.0	**9.39**	Normal	neg.	**Some T2-h. lesions**	n. d.	Normal	no
	2	4	n. d.	2.0	6.00	Normal	neg.	Unspecific	n. d.	n. d.	no
	3	1	1	0.7	2.89	Normal	n. d.	n. d.	n. d.	n. d.	no
	4	1	1	0.7	2.96	Normal	neg.	Normal	n. d.	n. d.	no
	5	1	1	0.7	2.87	Normal	n. d.	n. d.	Normal	n. d.	no
	6	1	1	2.7	4.01	Normal	n. d.	n. d.	n. d.	n. d.	no
	7	4	n. d.	3.7	**7.48**	Normal	n. d.	n. d.	Unspecific	n. d.	no
	8	1	1	0.7	2.81	Normal	n. d.	Normal	Normal	Normal	no
	9	1	1	1.7	7.53	Normal	n. d.	Unspecific	Normal	n. d.	no
	10	1	1	0.7	4.32	Normal	n. d.	n. d.	n. d.	n. d.	no
	11	1	1	1.0	6.0	Normal	n. d.	Unspecific	Normal	n. d.	no
	12	1	n. d.	3.0	4.26	Unspecific	n. d.	n. d.	n. d.	n. d.	no
	13	1	1	2.3	5.64	Normal	n. d.	Normal	n. d.	n. d.	no
	14	1	1	**31.7**	4.01	Normal	n. d.	Normal	Normal	Normal	no

*act., activated; CE, contrast enhancement; CC, CSF cell count per μl; cMRI, cerebral magnetic resonance imaging; inc., inconclusive; LETM, longitudinally extensive transverse myelitis; MRZ, intrathecal polyspecific antiviral immune response (positive MRZ reaction: 2 of 3 antibody indices against measles, rubella and/or varicella zoster virus are positive); n. d., not done/no data; ON, optic neuritis; Q-alb, quotient of albumin × 10^−3^ (marker of blood-brain barrier dysfunction); sMRI, spinal magnetic resonance imaging; T2-h. lesion, T2-hyperintense lesion; VEP, visual evoked potentials. Positive OCB are defined as pattern type 2/2a or type 3/3a. Negative OCB are defined as pattern type 1 or type 4. Bold entries indicate a pathology*.

Interestingly, all patients with OCB exclusively in tears had normal visual evoked potentials and CSF parameters (cell count, blood-brain barrier function, cell profile, MRZ reaction). Only one patient showed a pathological magnetic resonance imaging (for details see Tables 3A,B). One other patient had a clinically detectable ocular infection.

The sensitivity and specificity for OCB detection in tears for MS/CIS diagnosis is 41 and 50%, respectively. Nevertheless, as in CSF, there is a statistically significant difference in the occurrence of OCB in tears between the control group and MS patients (*P* = 0.0302).

## Discussion

This study likewise detected oligoclonal IgG in tear fluid. Compared to the positive studies of one research group with high agreement rates between CSF and tears of all CSF^OCB+^ patients [Forzy et al. (15): 73%; Devos et al. (16): 96%; Calais et al. (17): 66%; Lebrun et al. (19): 96%], our study found a significantly lower detection rate of OCB in tear fluid: 7 out of 18 (39%) analyzable MS patients (96% CSF^OCB+^) or 12 out of 26 analyzable CSF^OCB+^ patients (46%) had a concordance of OCB between cerebrospinal and tear fluid. The concordance for OCB absence or presence in all analyzable patients was 56% (24 of 43 patients). It is important to remember that in 59% of all samples with positive OCB in tears only a marginal OCB pattern was present.

How can these different study results be explained? All previous studies that investigated OCB in tear fluid differed considerably in the methodology used to (a) obtain tear fluid and (b) detect OCB (Table 1). Tear collection was performed using either glass capillary tubes or Schirmer strips. Tear production was partly stimulated by onions, ammonia, or warm air flow. Protein separation was performed on agarose or polyacrylamide gels by electrophoresis or isoelectric focusing. IgG was visualized by silver staining or immunoblotting. In the positive studies the detection of OCB in tear fluid, however, was achieved with all the different above mentioned procedures. In our study tear collection by Schirmer strips appeared to be the best reproducible collection technique whereas other collection methods (i.e., capillary with and/without flush) provided insufficient material for detailed analysis.

As a possible explanation for the discrepancy between the study results, the duration of tear collection was considered responsible in the most recent studies. To avoid dilution by reflex secretion, a maximum duration of 1 min for tear collection was suggested (15–17, 19). However, in these studies no threshold was mentioned concerning the required minimal running distance of tears on Schirmer strips. None of our patients had a lacrimation that would wet a Schirmer strip (35 mm) within 1 min. Only five patients (8%) moistened the complete Schirmer strip within 2 min. For a reliable usability of the sample, our study showed that the minimum running distance should be between 4 and 8 mm. Nevertheless, reliable results were only achieved from ~10 mm upwards. Accordingly, with the prerequisite of a maximum tear collection time of 1 min, hardly any patients could have been included in our study. In addition, in our study 13 patients with positive OCB in tears had a collection time of 5 min, which argues against the hypothesis of reflex dilution. An excessive dilution of the tear sample due to a longer collection time is also opposed by an average tear volume of 20 μl in our study. Devos and co-workers used a maximum of 30 μl (16).

Another relevant factor which interferes with tear OCB detection are tear specific protein bands. Accordingly, for a reliable analysis of OCB in tears an isolation of tear IgG by affinity chromatography is suggested. All previous studies analyzed crude tears. This certainly had an impact on the results.

If we propose that OCB in CSF should also be detectable in tears, in our study 14 of 26 CSF^OCB+^ patients (54%) and 10 of 17 MS patients (59%) would have “false negative” OCB in tears. Even in the CIS-study by Calais and co-workers, 15 of 44 CIS patients (34%) would have “false negative” tear results. Considering these fragile results and the high dropout rate of 22% of patients with poor tear production, which diminishes the practical applicability, we cannot recommend that tear OCB detection may replace CSF OCB detection. Moreover, one should also consider the gain in information due to CSF the differential diagnosis of MS and MS mimics (27).

The trigger for the production of OCB in tear fluid is not yet known. Our result of unique bands in tears suggests that in some cases there might be an independent local mucosal immune response. This aspect is supported by the second work by Coyle et al. who detected an independent OCB pattern in all six samples compared to serum and CSF (11). Moreover, in the studies by Martino et al. (14) one patient and by Devos et al. (16) four patients with unique OCB in tears without corresponding bands in serum or CSF were detected (14, 16). Both, the patient with zoster ophthalmicus (for details see Table 3A, Patient #4, OI disease group), and the patient with optic neuritis without CSF OCB (for details see Table 3A, Patient #17, RRMS disease group) suggest that ocular inflammation may lead to the presence of OCB in tears. This is supported by the significant relationship between visual evoked potential alterations and tear OCB positivity. Possibly OCB may develop in infectious or autoimmunological eye diseases in tear fluid. It would be worthwhile to test this hypothesis in a larger study with patients with acute and chronic eye diseases, as this could result in a possible new diagnostic tool for eye diseases and might provide new insights in the pathophysiology of eye diseases. Besides that, the occurrence of unique OCB in tears raises the controversy as how many of positive tear OCB results in MS patients are due to the chronic inflammatory CNS disease or to a present or previous inflammatory eye disease. Since the pattern between OCB in tears and CSF often do not coincide completely a different origin might be suggested.

In conclusion, OCB are detectable in tears. The high dropout rate of patients, the low concordance rate of OCB detection between CSF and tears in our study, and the ambiguous results in previous studies limit the application in daily clinical practice. Interestingly in 5 patients OCB were positive in tears but lacking in CSF. OCB detection in tears might be a useful test for ophthalmological diseases and differential diagnosis.

## Ethics Statement

This study was carried out in accordance with the recommendations of the ethics committee, Hannover Medical School. The protocol was approved by the local ethics committee (No. 7218), Hannover Medical School.

## Author Contributions

MH, LB, K-WS, SA, SG, and TS obtained the samples. MH, UW, LB performed the experiments. MH, UW, and MS conceived and designed the study. MH, UW, LB, K-WS, SA, SG, TS, and MS analyzed the data. MH, UW, TS, and MS wrote the paper. PS collected new samples, analyzed data and helped to extend the study according to the suggestion of the reviewers. All authors contributed to manuscript revision, read, and approved the submitted version.

### Conflict of Interest Statement

This research was partly supported by Merck KGaA Darmstadt, Germany. The funders had no role in data collection, analysis and interpretation of the results, decision to publish, or preparation of the manuscript. MH, UW, LB, PS, K-WS, and SG declare that they have no conflict of interest concerning this paper. K-WS received travel grants for scientific meetings from Merck, UCB Pharma and Alexion outside the submitted work. TS received honoraria for scientific lectures or consultancy from Alexion, Bayer Vital GmbH, CSL Behring, Merck, Novartis, and Sanofi outside the submitted work. MS has received honoraria for scientific lectures or consultancy from Alexion, Bayer HealthCare, Biogen, Baxalta/Shire, CSL Behring, Grifols, MedDay, Merck-Serono, Novartis, Roche, Sanofi-Genzyme, and Teva. His institution received research support from Bayer HealthCare, Biogen, Genzyme, Merck-Serono, Novartis, and Teva. PS received travel grants for scientific meeting from Merck. The remaining author declares that the research was conducted in the absence of any commercial or financial relationships that could be construed as a potential conflict of interest.
